# Synthesis and quality assessment of combined time-series and static medical data using a real-world time-series generative adversarial network

**DOI:** 10.1038/s41598-024-69812-7

**Published:** 2024-08-17

**Authors:** Jaewon Kim, Hyunwoo Choo, Soo-Yong Shin, Kyoung Doo Song

**Affiliations:** 1 Department of Digital Health, Samsung Advanced Institute for Health Sciences and Technology, Sungkyunkwan University, Seoul, Republic of Korea; 2 Department of Radiology, Samsung Medical Center, Sungkyunkwan University, 81 Irwon-Ro, Gangnam-Gu, Seoul, 06351 Republic of Korea

**Keywords:** Synthetic data, Generative adversarial networks, Data quality, Medical data, Sequential data, Privacy, Computational biology and bioinformatics, Medical research

## Abstract

This study addresses challenges related to privacy issues in utilizing medical data, particularly the protection of personal information. To overcome this obstacle, the research focuses on data synthesis using real-world time-series generative adversarial networks (RTSGAN). A total of 53,005 data were synthesized using the dataset of 15,799 patients with colorectal cancer. The results of the quantitative evaluation of the synthetic data’s quality are as follows: the Hellinger distance ranged from 0 to 0.25; the train on synthetic, test on real (TSTR) and train on real, test on synthetic (TRTS) results showed an average area under the curve of 0.99 and 0.98; a propensity mean squared error was 0.223. The synthetic and real data were similar in the qualitative methods including t-SNE and histogram analyses. The application of synthetic data in predicting five-year survival in colorectal cancer patients demonstrates comparable performance to models based on real data. This study employs distance to closest records and membership inference test to assess potential privacy exposure, revealing minimal risk. This study demonstrated that it is feasible to synthesize medical data, including time-series data, using the RTSGAN, and the synthetic data can be evaluated to accurately reflect the characteristics of real data through quantitative and qualitative methods as well as by utilizing real-world artificial intelligence models.

## Introduction

Medical data comprises various types, including basic patient information; clinical records generated during the treatment process, such as diagnoses, prescriptions, test results, and surgical records; time-series data, such as vital signs; and medical imaging data, such as plain radiography, CT scans, and MRIs. Recently, there has been an increase in research focused on developing medical artificial intelligence (AI) applications utilizing deep-learning techniques based on this extensive medical dataset. Training these deep-learning models requires a substantial volume of high-quality data. However, accessing and utilizing medical data presents unique challenges, predominantly owing to privacy concerns associated with the handling of sensitive patient information^1^.

Recently, generative models in deep learning have been actively researched, making it possible to create synthetic data that accurately reflect the characteristics of real data^2^. Consequently, instead of using real data that are difficult to disclose, training useful models using synthetic data generated by generative models has gained attention as a way to protect personal information^3,4^.

As generative models are a relatively new field of research, there is no established methodology for assessing the similarity between synthetic and real data. In the image domain, evaluation methods for synthetic data such as inception scores have been studied^5^. However, there is a lack of research on evaluation methods for tabular data, including time-series data. For such data, measures such as the Kullback–Leibler (KL) divergence^6^ or Jensen-Shannon divergence^7^ can be used to compare the probability distributions of real and synthetic data. However, if two probability distributions are generated in different ranges, the probability value in that range becomes zero and the KL divergence value cannot be obtained. Due to the nature of medical data, long-tailed phenomena may occur^8^, which may prevent the distributions of real and synthetic data from overlapping. In this case, the KL and the Jensen-Shannon divergences cannot be calculated. Therefore, a different evaluation method was required.

There has been a debate regarding the disclosability of synthetic data. Initially, it was believed that since synthetic data is randomly generated and closely mimics the characteristics of real data, it could be safely disclosed. However, recent discussions suggest that synthetic data may still align significantly with real data^9^. This revelation has heightened concerns around disclosure risk, which involves the potential for sensitive or identifiable information to be deduced from the release or exposure of such data^10^.

The aim of this study was to (1) synthesize medical data using a real-world time-series generative adversarial network (RTSGAN)^11^, a GAN-based generation model; (2) evaluate the quality of the synthetic data quantitatively and qualitatively; (3) apply the synthetic data to medical AI models and compare their performance with the results of real data to assess the feasibility of utilizing synthetic data; and (4) assess the disclosure risk of synthetic data.

## Results

A total of 53,005 data were synthesized using RTSGAN.

### Similarity between synthetic and real data

**Figure 1:**
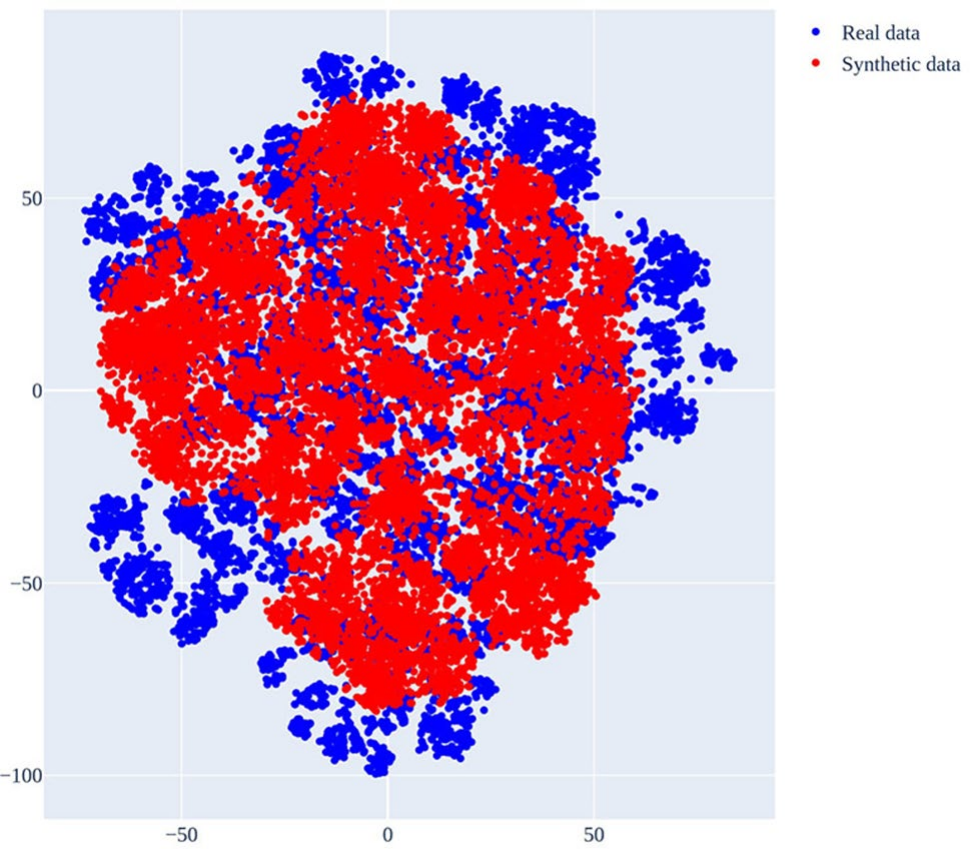
t-SNE results. Blue shows the t-SNE for hidden on real data and red shows the t-SNE for hidden on synthetic data.

The Hellinger distances for the dynamic and static variables between the real and synthetic data are listed in Tables 1 and 2, respectively. The results for train on synthetic, test on real (TSTR) and train-on-real, test on synthetic (TRTS) showed that the mean area under the curve (AUC) values were 0.99 and 0.98, respectively. The propensity mean square error (MSE) was 0.223 (standard deviation = 0.08). The results of t-distributed stochastic neighbor embedding (t-SNE), a qualitative method, showed that the real data and synthetic data were similar (Fig. 1). The dispersion value of t-SNE was lower in the synthetic data compared to the real data: the dispersion values for t-SNE 1 and t-SNE 2 were 1396.5 and 2010.9 in the real data, and 1023.3 and 1500.7 in the synthetic data. From the histogram analysis, it was also observed that the synthetic and real data were similar. The histograms for each variable are shown in the Appendix figures.

**Table 1:** Hellinger distance for dynamic variables.

Variables	Hellinger distance
CEXM_RSLT_CONT	0.000437
TIMESTAMP	0.023038
SGPT_SRMV_LN_CNT	0.058716
SGPT_MTST_LN_CNT	0.014385
LINE	0.004744
CSTR_END_YMD	0.141307
CSTR_NT	0.122393
RDT_END_YMD	0.089232
BPTH_BPSY_RSLT_CONT	0.0033
IMPT_HM1E_RSLT_CD	0
IMPT_HS2E_RSLT_CD	0
IMPT_HS6E_RSLT_CD	0
IMPT_HP2E_RSLT_CD	0
MLPT_KRES_RSLT_CD	0.101
MLPT_NREX_RSLT_CD	0.1193
MLPT_BRME_RSLT_CD	0.106
SGPT_PATL_STAG_VL	0.0759
SGPT_PATL_T_STAG_VL	0.0913
SGPT_PATL_N_STAG_VL	0.0733
SGPT_PATL_M_STAG_VL	0.0372
SGPT_TUMR_BUDD_CD	0.0279
OPRT_CLCN_OPRT_KIND_CD	0.0516
OPRT_CURA_RSCT_CD	0.0642
CSTR_PRPS_CD	0.0217
CSTR_REGN_CD	0.0884

**Table 2:** Hellinger distance for static variables.

Variables	Hellinger distance
BSPT_FRST_DIAG_YMD	0.135982
BSPT_DEAD_YMD	0.246331
BSPT_IDGN_AGE	0.007067
BSPT_SEX_CD	0.0059
BSPT_FRST_DIAG_CD	0.0348
BSPT_STAG_VL	0.0623
BSPT_T_STAG_VL	0.0722
BSPT_N_STAG_VL	0.0385
BSPT_M_STAG_VL	0.0241

### Comparison of performance for the models using synthetic data and real data

**Figure 2:**
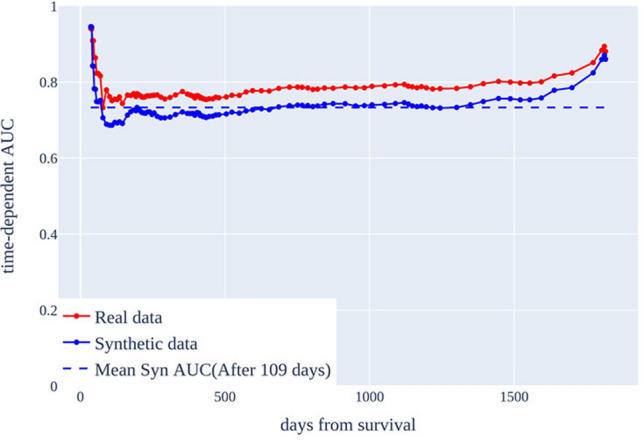
Comparison of AUC over time between real and synthetic data. The x-axis represents the survival time and the y-axis represents the AUC value by time-of-day. The time-of-day AUC of the real data is in red and the time-of-day AUC of the synthetic data is in blue.

**Figure 3:**
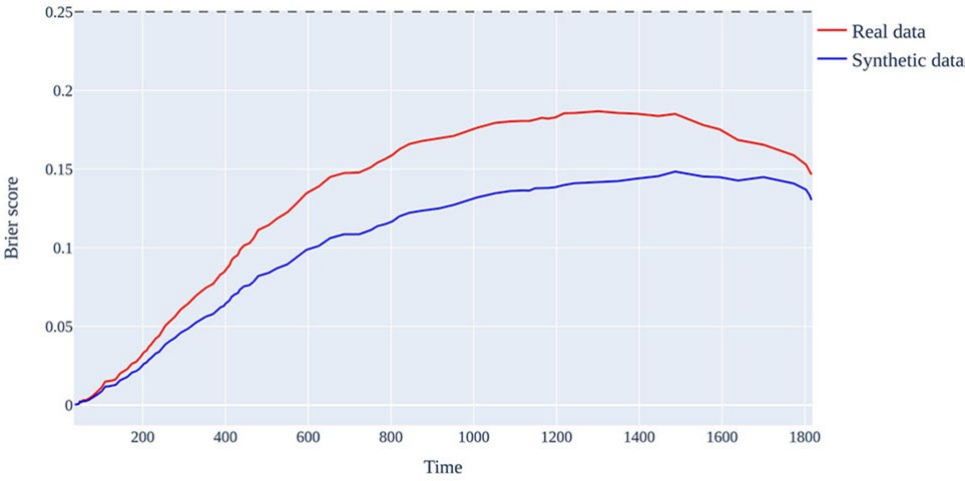
Brier score comparison of real and synthetic data. The x-axis represents time-of-day and the y-axis represents Brier score. Brier scores using real data are red and those using synthetic data are blue.

We trained the healthcare AI model to predict 5-year survival using real and synthetic data and compared their performance. The results are summarized in Table 3. The AI model trained with real data exhibited a higher C-index than that trained with synthetic data. The opposite was true for the Brier score and IBS. We also examined the metric results for each time point, in addition to the overall metrics of the model. First, we plotted the AUC values for predictions at each time point based on the C-index using real and synthetic data, as shown in Fig. 2. We observed that the performance with real data was generally better than that with synthetic data but in a relatively similar fashion. We also examined the Brier score over time using real versus synthetic data. At every time point, the synthetic data outperformed the real data in terms of Brier score (Fig. 3).

**Table 3:** Survival prediction results of models trained real and synthetic data.

Metric	Real data	Synthetic data
C-Index	0.777	0.742
Brier score	0.098	0.075
IBS	0.138	0.107

### Assessment of disclosure risk of synthetic data

To assess the disclosure risk, we separate the training data used for data synthesis into target data (T), test data for holdout data (H), and synthesized data for synthetic data (S)^12^. We calculate the distance of the nearest data (DCR) from target data (T) using NearestNeighbors^13^ in sklearn.neighbors, and apply One-Hot-Encoding for categorical variables. The DCR evaluation shows that the minimum distance between target data (T) and holdout data (H) is 2.45, and the minimum distance between target data (T) and synthetic data (S) is 3.46. Although these are close to zero, indicating that the two data are in perfect accordance, we see that the minimum distance between the real data, target data(T) and holdout data(H), is smaller than the minimum distance between target data(T) and synthetic data(S).

We also performed inference test (MIT)^14,15^ and maintained a 1:1 ratio of real data to test data for attack model evaluation. To achieve this, the number of real data and test data used in synthesis was adjusted to 1:1, and the number of synthetic data used in the shadow model was adjusted to the same ratio. For the label classification model, we used support vector machine model^16^. The result of MIT was an accuracy of 0.499.

## Discussion

We synthesized data from patients with colorectal cancer using RTSGAN. Although, previous synthetic healthcare data research focused on synthesizing single tables or time-series data, we synthesized data composed of multiple tables simultaneously by joining multiple event tables to the patient demographic table, which is a distinct approach from previous studies. Then, we conducted both quantitative and qualitative assessments of the synthetic data to gauge their quality. Additionally, we evaluated the practical utility of the synthetic data by applying them to real-world predictive models.

For the quantitative method, we first examined the Hellinger distance for each variable in both the real and synthetic data. Except for a few numerical variables, most Hellinger distances were below 0.1, suggesting that the distributions of the real and synthetic data were similar. For TSTR and TRTS, the average AUC values are 0.99 and 0.98, respectively, indicating that synthetic data can effectively replace real data for model training. The propensity MSE is 0.223, which, being close to zero, implies that from a model's perspective, synthetic data are indistinguishable from real data. These methods evaluate the distributional similarity between real and synthetic data, assess the model's performance using both data types, and verify whether the synthetic data capture the overall characteristics of the real data and whether the model can differentiate between the two. These are crucial for quantitatively ascertaining the quality of synthetic data generation.

For qualitative analysis, we used histograms and t-SNE analyses. We compared the histograms of real and synthetic data variable by variable and then applied t-SNE to the hidden data generated during model training. This was done to check whether the two datasets corresponded closely to the lower dimensions, and we determined that they were similar. Although these qualitative methods do not provide a quantitative measure of similarity, they are valuable for verifying the initial quality of synthetic data generation.

Most variables in the synthetic data reflect the characteristics of the real data well. However, there are discrepancies between the synthetic and real data for variables such as the first diagnosis date, date of death, anticancer treatment end date, and number of chemotherapy treatments. These variables include low-frequency values or extreme values in the real data, which the RTSGAN model we used struggles to generate accurately. Additionally, when the real data exhibit multiple patterns, RTSGAN seems to focus on the dominant pattern, potentially overlooking other less common patterns. This is likely the reason for the observed discrepancies between the synthetic and real data in some variables. Furthermore, the dispersion of synthetic data in the t-SNE plot is lower than that of the real data, which can also be attributed to the characteristics of RTSGAN in data synthesis.To evaluate the usefulness of the synthetic data, we trained a medical AI model using both real and synthetic data and then assessed their performance using validation data. The C-index values for the models trained with the real and synthetic data were 0.777 and 0.742, respectively. The AUCs of the predictions at each time point were compared based on the C-index. Although the model generally performed better with real data, the difference was marginal, with an AUC root-mean-square error of 0.002. Notably, the model performed better with synthetic data, particularly before the 42-day mark. In the case of synthetic data, from day 686 onwards, the AUC values were typically similar to or exceeded the mean value, except for data before day 109, where there was a significant deviation from the synthetic data. This indicates that the model was effective in predicting survival probabilities in the short, mid, and long terms.

We also evaluated the models' performance over time using the Brier score, which ranged between 0 and 0.25, with 0.25, representing complete failure of the model's predictions. For models trained with real and synthetic data, the average Brier scores for real and synthetic data were 0.098 and 0.072, respectively, at each time point, and neither exceeded 0.25. This supports the feasibility of using synthetic data in colon cancer scenarios. Notably, training with synthetic data consistently resulted in lower Brier scores compared with real data, with the largest disparity observed at 1,218 days after the first visit. For the model trained with real data, the Brier score was 0.1854, indicating an approximately 18.5% chance of incorrect prediction, whereas for synthetic data, the Brier score was 0.1399, reflecting an error probability of approximately 14%. These findings suggest that from a Brier score perspective, training with synthetic data yields better performance than training with real data. Additionally, the IBS values for the real and synthetic data were 0.138 and 0.107, respectively, indicating higher overall prediction accuracy when the model was trained with synthetic data. This demonstrates the realistic nature of synthetic data and their effective application in real-world medical scenarios. By comparing the outcomes of using synthetic data in a healthcare AI model with those from real data, we can confirm the practical utility of synthetic data while still adhering closely to the characteristics of real data.

The synthetic data we generated for colorectal cancer included 33 variables. This complexity reduces the likelihood of creating synthetic data that exactly matches the actual data. In fact, there were no instances where the synthetic data and the real data matched completely in our study. To assess the risk of personal information exposure, we used the DCR value. We compared the minimum distance between the target data (T) and the holdout data (H), and between the target data (T) and the synthetic data (S). These distances were found to be 2.45 and 3.46, respectively. The critical observation is that the distance between the target data (T) and synthetic data (S) is larger than that between the target data (T) and the holdout data (H). This suggests that, from a privacy perspective, the synthetic data is actually preferable as it poses a lower risk of revealing personal information. In addition, we also performed MIT to check whether the synthetic data was included in the real data. In membership inference attacks, the ideal accuracy is 50%, which is the baseline accuracy at which the attacker randomly guesses whether a record belongs to the training data set of the target model. In this paper, we found that the accuracy of MIT was 49.9%, which is close to the ideal accuracy, indicating that the synthetic data is unlikely to be included in the real data.

In conclusion, this study demonstrated that it is feasible to synthesize medical data, including time-series data, using the RTSGAN, and the synthetic data can be evaluated to accurately reflect the characteristics of real data through quantitative and qualitative methods as well as by utilizing real-world artificial intelligence models.

## Methods

The study protocol was in accordance with the ethical guidelines of the 1975 Declaration of Helsinki. This retrospective study was approved by the Institutional Review Board of Samsung Medical Center (IRB file No. SMC 2021-02-128). The requirement for an informed consent was waived by the ethics committee of Samsung Medical Center because the study involved a retrospective review of anonymized medical data.

### Data sets

**Figure 4:**
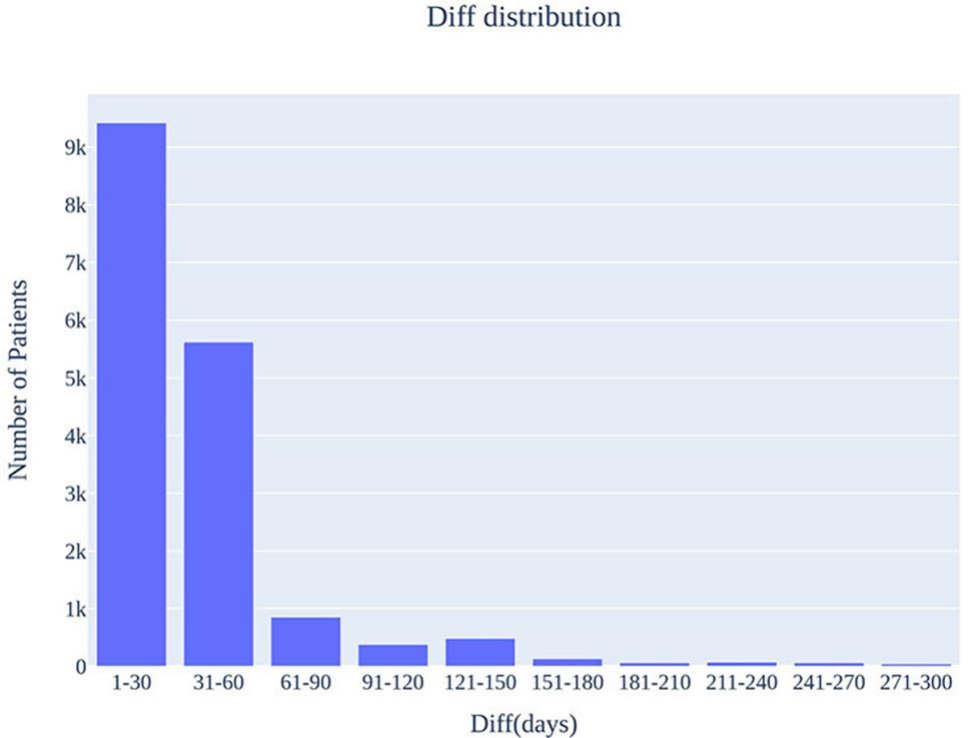
Diff distribution (bin size = 1 month). The x-axis represents the Diff value, and the y-axis represents the number of patients.

In this study, the real data used for data synthesis consisted of records of patients with colorectal cancer who received their initial treatment at the Samsung Medical Center from January 1, 2008, to July 31, 2021. During this period, 30,527 patients with colorectal cancer were registered at the Samsung Medical Center. The total patient population included those who initially received treatment at outside institutions and were subsequently transferred to the Samsung Medical Center. However, our data did not include records of treatments received at other institutions. Therefore, to select only patients who received the initial treatment at the Samsung Medical Center, the following process was undertaken. First, we calculated the “Diff” value, which is defined as [Min (date of first surgery, date of first anti-cancer treatment)—date of first diagnosis]. The distribution of the “Diff” value among all patients is shown in Fig. 4. Next, we investigated the number of patients selected when the Diff value was at three months and six months (Table 4). Typically, treatment begins within three months of diagnosis. Therefore, after consultation with clinical experts, we decided on a three-month cutoff for the Diff value to select the study subjects. As a result, out of 30,527 raw data, 15,899 patients with a Diff value of three months or less were selected.

**Table 4:** Selection of subjects according to Diff value cutoff.

Cutoff of Diff^a^	Total	Target	≥ cutoff	< 0	Null
3 months	30,527	15,899	2,231	14	12,383
6 months	30,527	16,878	1,252	14	12,383

^a^Diff: Min (first surgery date, first anti-cancer treatment date)—first diagnosis date.

After screening the collected real data, we identified data errors that did not align with clinical scenarios. We applied logic based on actual clinical situations to exclude these erroneous data. By omitting such erroneous data before synthesis, we enhanced the quality of synthetic data. The logic applied is as shown in Appendix Textbox 1. Finally, a real dataset comprising 15,799 individuals was constructed for data synthesis. The basic statistics of the real data group are shown in Table 5.

**Table 5:** Demographics of real data group.

Variables	Patients (n = 15,799), n (%)
Age Group (years)	
< 18	3(0.02)
18–29	117(0.74)
30–39	623(3.94)
40–49	1997(12.64)
50–64	7048(44.61)
≥ 65	6011(38.05)
Gender	
Male	9062(57.4)
Female	6737(42.6)
Tumor location	
Rectosigmoid junction	9142(57.9)
Colon	5164(32.6)
Rectum	1493(9.5)
Death	
Survived	13015(82.2)
Died	2784(17.8)

### Preprocessing and dataset construction for data synthesis

The National Cancer Center, a public institution in South Korea, built a platform called CONNECT, which contains standardized cancer big data from medical institutions for research purposes. Real-world data were collected according to the data format of the CONNECT platform. In the CONNECT platform, the data are in a multi-table format, divided into separate tables for static patient baseline information that does not change over time, and dynamic information with specific occurrence times, such as examinations, pathology, surgeries, chemotherapy, and radiation therapy. The details of these tables are provided in Appendix Table 1. Considering their sequential relationships, a process was undertaken to convert the date variables into numerical ones for synthesis. This process is described in Appendix Textbox 2. We then joined other event tables based on patient basic information tables whose values did not change over time, such as the first diagnosis date or sex, to generate consolidated data on how many days had passed since the first diagnosis date the patient had an event.

### Data synthesis

**Figure 5:**
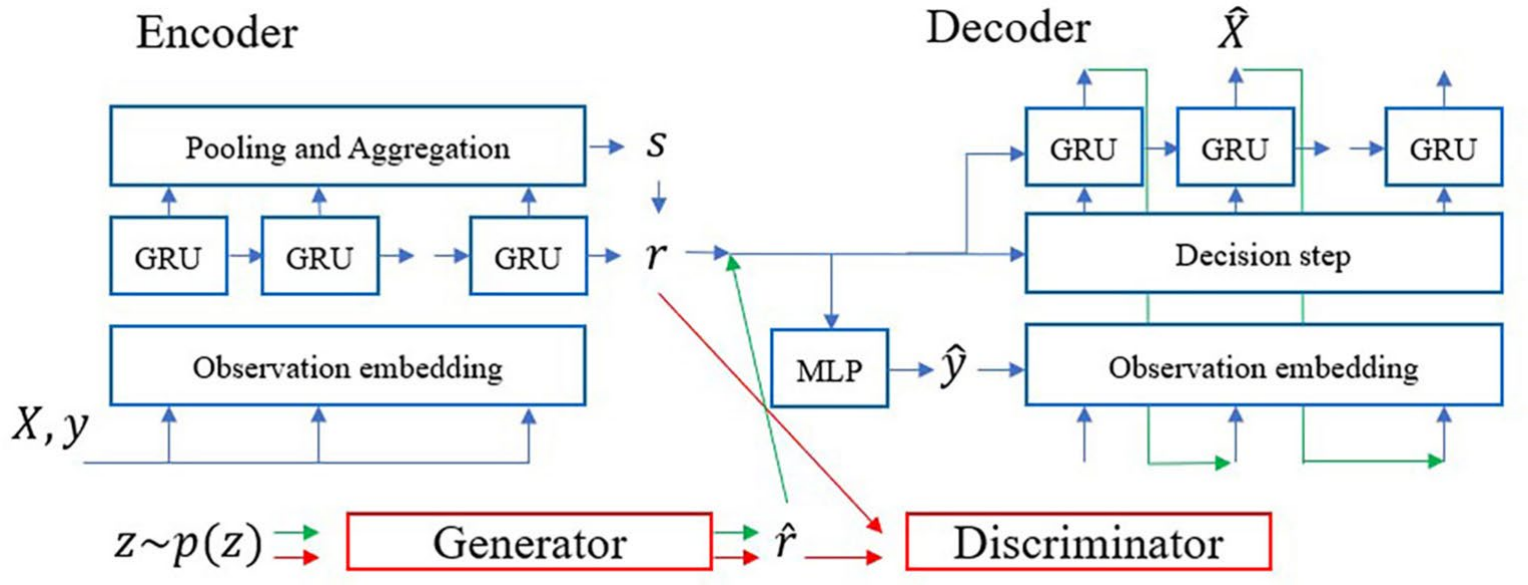
Architecture of the RTSGAN. The blue line represents the learning process of the encoder–decoder module, the red line represents the learning process of the generation module, and the green line represents the process of generating synthetic data after learning.

In this study, the RTSGAN was used to synthesize medical data with irregular time intervals and variable time lengths. The RTSGAN is a model specialized for medical data synthesis with irregular data generation cycles and consists of an encoder–decoder module and a generation module (Fig. 5). The detailed description of RTSGAN is provided in the Appendix Textbox 3. The main parameters used to train the model are listed in Appendix Table 2.

### Evaluation of quality of synthetic data

Quantitative and qualitative evaluations were conducted to ensure that the synthetic data were accurately generated. Furthermore, we applied the synthetic data to a real medical AI model to evaluate its performance and confirm the feasibility of utilizing synthetic data.

Quantitative methods include the Hellinger distance; TSTR; TRTS; and propensity MSE. The Hellinger distance is used to numerically determine how similar the two probability distributions are and is calculated based on the Bhattacharyya coefficient, which is similar in nature^17^. The Hellinger distance has a value of 0 when the two probability distributions match and a value closer to 1 when the two probability distributions do not match. The calculation method of the Hellinger distance is described in detail in the Appendix Textbox 4.

TSTR is a method of training a classifier model with synthetic data and validating it using real data. A comparison is made between the model's metric on the train data and the model's performance on the test data^18^. If synthetic data are well-generated and follow the characteristics of real data, the model will behave normally when real data are applied to a model trained with synthetic data, and the difference between the two performances will be small. Conversely, in TRTS, the classifier model is trained with real data and validated with synthetic data. The problem with this method is that, even if a mode collapse phenomenon appears in the synthetic data, it does not affect the TRTS evaluation method. However, the WGAN-GP^19^, which was used in the generation module of this study, improved the mode collapse problem; therefore, we also verified TRTS.

Propensity MSE is a method that generates training and test data by labeling real and synthetic data, mixing them in a 1:1 ratio, and then training and testing a model to classify them to measure how well it can distinguish between real and synthetic data^20^. In the case of propensity MSE, it had a value between 0 and 0.25; however, in this study, scaling was performed to make it easier to interpret, and as a result, it was converted to have a value between 0 and 1 as follows:

$$propensity MSE(Scaled)= \frac{1}{N}\sum_{i}^{N}\frac{{({p}_{i}-0.5)}^{2}}{0.25}$$

where N is the size of the dataset and p is the model-generated pseudo probability for each sample. If the classification model does not distinguish between real and synthetic data, the propensity MSE converges to zero.

Qualitative evaluation methods included histogram and t-SNE methods. Histograms were used to examine the distribution of data and compare the distribution between the real and synthetic data. Although it cannot provide a numerical representation of the similarity between real and synthetic data, it can provide the first indication that the probability distributions of the two datasets are similar. t-SNE is a dimensionality reduction method for high-dimensional complex data to low-dimensional data and is characterized by close correspondence between similar data in low dimensions^21^. Considering these features, if the synthetic data are well-generated, the t-SNE for the synthetic data should not be significantly different from that for the real data.

### Application of synthetic data to a real-world medical AI model

We applied synthetic data to a real-world medical AI model to evaluate its usefulness. After applying real and synthetic data to a medical AI model, the quality of the synthetic data can be evaluated by comparing the similarity of the results obtained using the synthetic data with those obtained using the real data. As a medical AI model, we used a random survival forest^22^ that predicts five-year survival using colon cancer patient data; the survival model's evaluation indicators include the C-index, Brier score, and integrated Brier score (IBS)^23–25^. Details about the C-index, Brier score, and IBS are described in the Appendix Textbox 5.

### Evaluation of disclosure risk of synthetic data

DCR is a method for assessing the likelihood of personal information exposure by measuring the distance between synthetic data and real data, typically measuring the distance from each synthetic data to the nearest real data^26^. The closer the distance between the data is to zero, the more similar the synthetic data is to the real data, which means the higher the probability of personal information disclosure.

The MIT is a method of inferring whether a particular data point belongs to the training dataset^14,15^. It consists of a target model that is trained with the actual training dataset, an attack model that inferred whether a particular data point belongs to the training dataset, and shadow training that is used to train the attack model. Shadow training, which is used to train a dual attack model, is based on the idea that training two models on similar datasets using the same model structure increases the likelihood that the two models will make similar predictions. If a shadow model trained on synthetic data that resembles real data learns to infer the presence of data points that contain the information the attacker wants to extract, an attack model can be created that successfully inferred data points that also exist in the target model's real training data. In other words, the attack model can analyze the shadow model's predictions to determine whether a particular data point belonged to the target model's training dataset. As a result, if the attack model can accurately distinguish whether a certain data point belongs to the target model's train data, it can infer that the data point is likely to belong to the target model's training dataset. In this paper, we use the membership inference test to verify whether the synthetic data belongs to the real data used to train the model.

### Supplementary Information

Supplementary Information.

## Data Availability

The synthetic datasets generated during the current study are available from the corresponding author on reasonable request. Our code and all results are available at https://github.com/bmiskkuedu/synthetic_cancer_patients.
