# Supplementary material for: Synthesis and quality assessment of combined time-series and static medical data using a real-world time-series generative adversarial network
Source: Sci Rep. 2024 Aug 17;14:19064. doi: 10.1038/s41598-024-69812-7 (PMC11330441; doi:10.1038/s41598-024-69812-7)
Supplement: Supplementary file 1 — Supplementary Information. [file 41598_2024_69812_MOESM1_ESM.pdf]

Synthesis and Quality Assessment of Combined Time-Series and Static Medical Data Using a Real-world Time-Series Generative Adversarial Network

Jaewon Kim[1], Hyunwoo Choo[1], Soo-Yong Shin[1], PhD, Kyoung Doo Song[1,2], MD, PhD

Multimedia Appendix

Appendix Table 1: List of colorectal cancer synthetic variables

Main Category	Table name	Table description	Variable name	Variable description
Examination	CLRC_PT_BSNF	Colorectal cancer_patient_basic information	PT_SBST_NO	Patient replacement number
			BSPT_SEX_CD	Patient gender code
			BSPT_IDGN_AGE	Patient age
			BSPT_FRST_DIAG_YMD	Date of first diagnosis
			BSPT_FRST_DIAG_CD	Diagnosis code
			BSPT_DEAD_YMD	Date of death
			BSPT_STAG_VL	Patient staging value
			BSPT_T_STAG_VL	Patient staging value_T
			BSPT_N_STAG_VL	Patient staging value_N
			BSPT_M_STAG_VL	Patient staging value_M
Pathology	CLRC_EX_DIAG	Colorectal cancer_diagnosis	PT_SBST_NO	Patient replacement number
			CEXM_RSLT_CONT	Diagnostic test results
			TIMESTAMP	Occurrence date
	CLRC_PTH_BPSY	Colorectal cancer_pathology_biopsy	PT_SBST_NO	Patient replacement number

			BPTH_BPSY_RSLT_CONT	Biopsy results
			TIMESTAMP	Occurrence date
	CLRC_PTH_MNTY	Colorectal cancer_Pathology_Immunity	PT_SBST_NO	Patient replacement number
			IMPT_HM1E_RSLT_CD	HM1E result code
			IMPT_HS2E_RSLT_CD	HS2E result code
			IMPT_HS6E_RSLT_CD	HS6E result code
			IMPT_HP2E_RSLT_CD	HP2E result code
			TIMESTAMP	Occurrence date
	CLRC_PTH_MLCR	Colorectal cancer_pathology_molecular	PT_SBST_NO	Patient replacement number
			MLPT_KRES_RSLT_CD	KRASMUTATION result code
			MLPT_NREX_RSLT_CD	NRASMUTATION result code
			MLPT_BRME_RSLT_CD	BRAFMUTATION result code
			TIMESTAMP	Occurrence date
	CLRC_PTH_SRGC	Colorectal cancer_Pathology_Surgery	PT_SBST_NO	Patient replacement number
			SGPT_PATL_STAG_VL	staging value
			SGPT_PATL_T_STAG_VL	staging value_T
			SGPT_PATL_N_STAG_VL	staging value_N
			SGPT_PATL_M_STAG_VL	staging value_N
			SGPT_SRMV_LN_CNT	resected lymph nodes
			SGPT_MTST_LN_CNT	metastatic lymph node
			SGPT_TUMR_BUDD_CD	Surgical resection surface distal cancer tissue

				size status code
			TIMESTAMP	Occurrence date
Surgery	CLRC_OPRT_NFRM	Colorectal cancer_surgery_information	PT_SBST_NO	Patient replacement number
			OPRT_CURA_RSCT_CD	Radical resection code
			OPRT_CLCN_OPRT_KIND_CD	Colorectal cancer surgery type code
			TIMESTAMP	Occurrence date
Treatment	CLRC_TRTM_CASB	Colorectal cancer_treatment_chemotherapy	PT_SBST_NO	Patient replacement number
			CSTR_REGN_CD	Anticancer drug treatment code
			CSTR_NT	Number of chemotherapy treatments
			CSTR_END_YMD	Anticancer drug treatment end date
			CSTR_PRPS_CD	Anticancer drug treatment purpose code
	CLRC_TRTM_RD	Colorectal cancer_treatment_radiation	PT_SBST_NO	Patient replacement number
			RD_STRT_YMD	Radiation treatment start date
			RD_END_YMD	Radiation treatment completion date

Appendix Table 2: List of parameters used in RTSGAN

Parameters	Descriptions	Value
epochs	Number of full passes through training set for autoencoder	800
iterations	Number of iterations through training set for WGAN	15000
d-update	Discriminator updates per generator update	5
Python-seed	Random seed of Python and NumPy	42
AE-batch-size	Minibatch size for autoencoder	128
GAN-batch-size	Minibatch size for WGAN	512
Embed-dim	Dim of hidden state	512
hidden-dim	Dim of GRU hidden state	128
layers	Number of layers	3
Ae-lr	Autoencoder learning rate	1e-3
Gan-lr	GAN learning rate	1e-4,
Noise-dim	Dim of WGAN noise state	512

Appendix Textbox 1: Description of data exceptions

1. Exclude (T stage !=3 & N stage !=0) when stage =3 in the patient basic information table.
2. Exclude cases with M stage : 1 and stage !=4 in the patient demographic table.
3. When lymph nodes are listed, exclude if metastatic lymph nodes are not listed.
4. When resected lymph nodes are 1~3, exclude if N stage != 4
5. Excluded patients with only patient basic information table values

Appendix Textbox 2: Description of date related variable processing

1. Date of birth: The date of birth of all patients was converted to a numerical value by subtracting the reference date (1900-01-01). The patient age was then calculated and converted.
2. First diagnosis date: The minimum value of the first diagnosis dates of all patients was designated as the reference date, and then converted to a numerical value indicating how many days after the reference date the first diagnosis was made.
3. Date of death: converted to a numerical variable indicating how many days after the first diagnosis date the patient died. Then we created and added a death status variable based on the presence or absence of the death date variable.
4. Variable related to the date of medical treatment: For each patient, we converted it into a numerical variable indicating how many days after the date of first diagnosis the medical treatment took place.

Appendix Textbox 3: RTSGAN

In this study, the RTSGAN was used to synthesize medical data with irregular time intervals and variable time lengths. The RTSGAN is a model specialized for medical data synthesis with irregular data generation cycles and consists of an encoder–decoder module and a generation module . The encoder–decoder module extracts features from the data into a fixed-dimensional latent space and the generation module synthesizes the extracted features. First, medical data consist of a dynamic variable X, whose value changes over time, and a static variable y, which does not change regardless of time, and are characterized by a very high missing rate for each variable because tests are performed according to symptoms. This makes it difficult for the model to learn information from data. Therefore, the encoder first performs observation embedding by considering not only the dynamic variable X and static variable y, but also the observation value of the previous time and the time information of when it was observed. Subsequently, through the N-layer gated recurrent unit (GRU) of the encoder, the value of s is obtained by utilizing the hidden layer of the GRU to effectively represent the properties of the time series during the pooling and aggregation processes. Subsequently, s is added to the last hidden state to obtain a latent vector r of fixed length. Next, the decoder recovers the dynamic variable $\hat{X}$. In a special case, it first obtains the static variable $\hat{y}$ recovered by the MLP layer and then utilizes it to recover the dynamic variable $\hat{X}$. At this time, the decoder's decision step determines which data will be observed at which point in time, and then restores the values based on that information through a stacked RNN (GRU) to complete the training of the encoder–decoder module. Subsequently, in the generation module, the latent vector r obtained through the encoder–decoder module is synthesized using a

GAN (WGAN-GP) to obtain $\hat{r}$. Finally, $\hat{r}$ generated by the generation module is used as an input to the previously trained decoder to generate synthetic data.

The main parameters used to train the model were epochs in the encoder–decoder module and iterations in the generation module; d-update, which is the ratio of Generator to Discriminator updates; hidden-dim, which is the dimensionality of the hidden dimension; and Ae-lr and Gan-lr, which determine the learning rate of the encoder–decoder module and the generation module.

Appendix Textbox 4: The Hellinger distance

The Hellinger distance is used to numerically determine how similar the two probability distributions are and is calculated based on the Bhattacharyya coefficient, which is similar in nature. First, the Bhattacharyya coefficient for the two probability distributions P and Q can be calculated as follows:

$$BC(P,Q) = \sum_{i=1}^{n} \sqrt{p_i q_i}$$

where n denotes the size of the sample space. The Bhattacharyya coefficient is characterized by the fact that the more similar the two probability distributions are, the closer they are to 1. Based on this, the Hellinger distances of the two probability distributions P and Q can be calculated as

$$H(P,Q) = \sqrt{1 - BC(P,Q)}$$

The Hellinger distance has a value of 0 when the two probability distributions match and a value closer to 1 when the two probability distributions do not match. Unlike the KL divergence, which has a larger value, the two distributions are different, and the Hellinger distance has the advantage of being easy to interpret because it shows the degree of similarity between two probability distributions as a value between 0 and 1.

Appendix Textbox 5: C-index, Brier score, and Integrated Brier score.

The C-index is the ratio of the sum of the number of samples listed in ascending order of survival time to the sum of the number of samples that survived longer than each observed event, and the sum of the number of samples listed in ascending order of predicted survival time to the sum of the number of samples that were correctly predicted to survive longer than each observed event. It is calculated as follows:

$$c = \frac{\sum_{i\in U}\left\{\sum_{T_j>T_i} 1_{f_j>f_i}\right\}}{\sum_{i\in U}\left\{\sum_{T_j>T_i} 1\right\}}$$

Where a large U is a set of uncensored data, T_i is the observed survival time of sample i and f_i is the predicted survival time of sample i and $1_{a>b}$ is 1 if a>b; otherwise, it is 0. The C-index has a value between 0 and 1, with values closer to 1 interpreted as accurate predictions and values closer to 0.5, as randomized predictions. The Brier score was used to evaluate the accuracy of the predicted survival function for a given time t and was calculated as follows:

$$Brier\ score = \frac{1}{n}\sum_{i=1}^{n}(p_i - O_i)^2$$

where p_i denotes the probability of an event predicted by the model for the ith subject and O_i denotes the observed outcome of the ith subject. IBS is the integral of the Brier score over time and is used to determine the overall performance of the model over all time bins It is calculated as follows:

$$IBS = \frac{1}{t_{max}}\int_0^{t_{max}} BS(t)dt$$

Appendix Figures: Histogram for each variable between the real and synthetic data

The following are the Histogram for each variable between the real and synthetic data. Blue shows the real data's histogram and red shows the synthetic data's histogram.

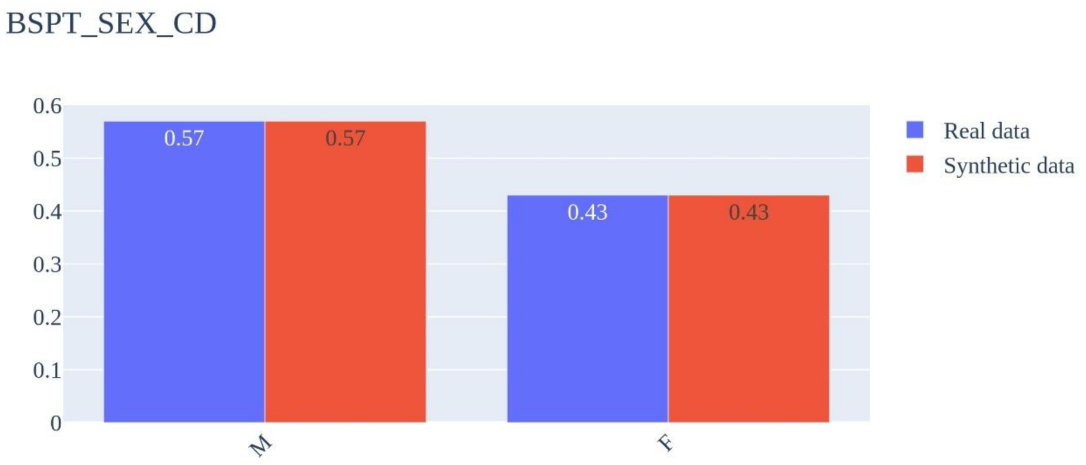

Figure 1: Histogram of basic patient gender code between real and synthetic data

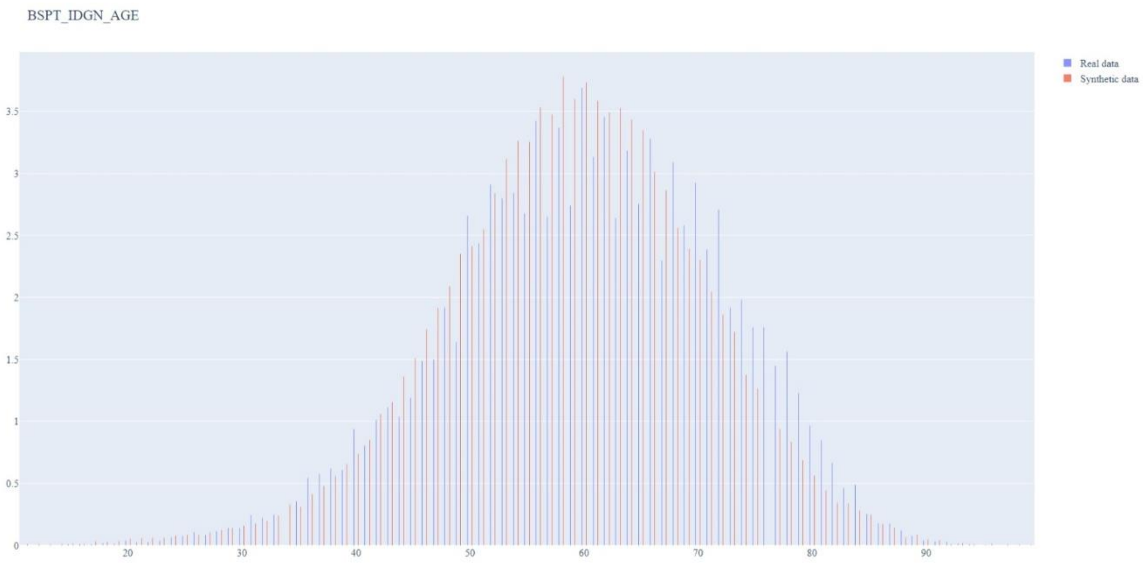

Figure 2: Histogram of basic patient age between real and synthetic data

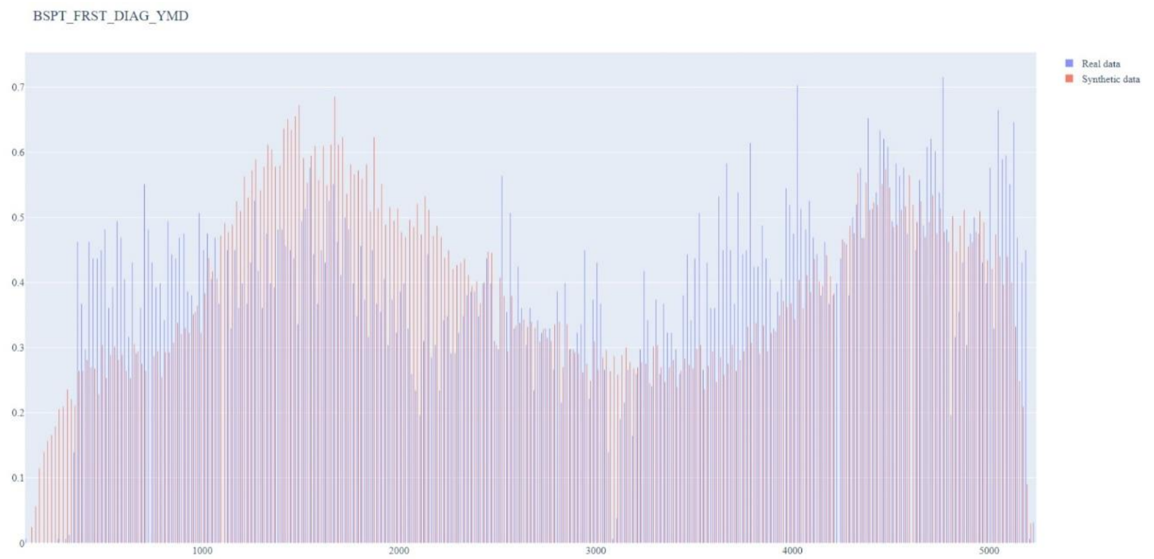

Figure 3: Histogram of first diagnosis date between real and synthetic data

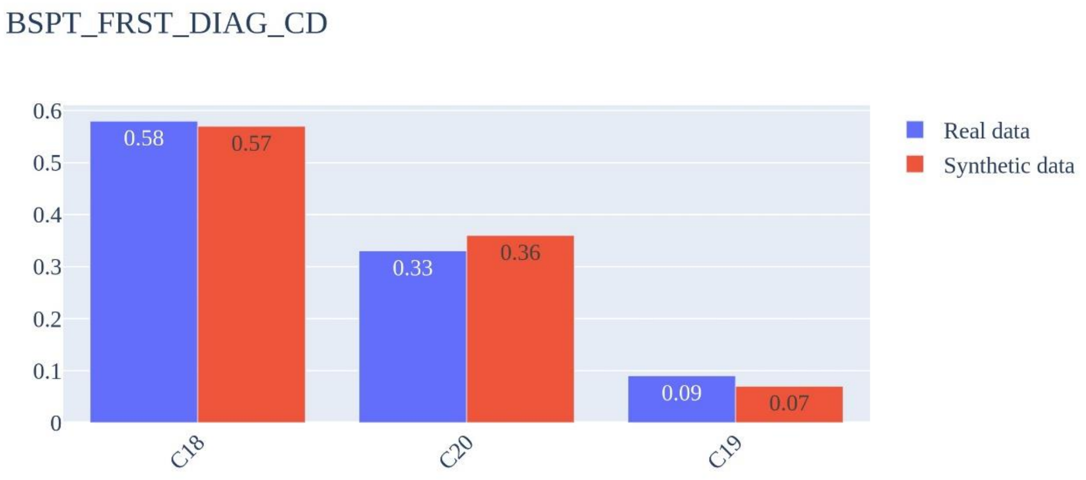

Figure 4: Histogram of diagnosis code between real and synthetic data

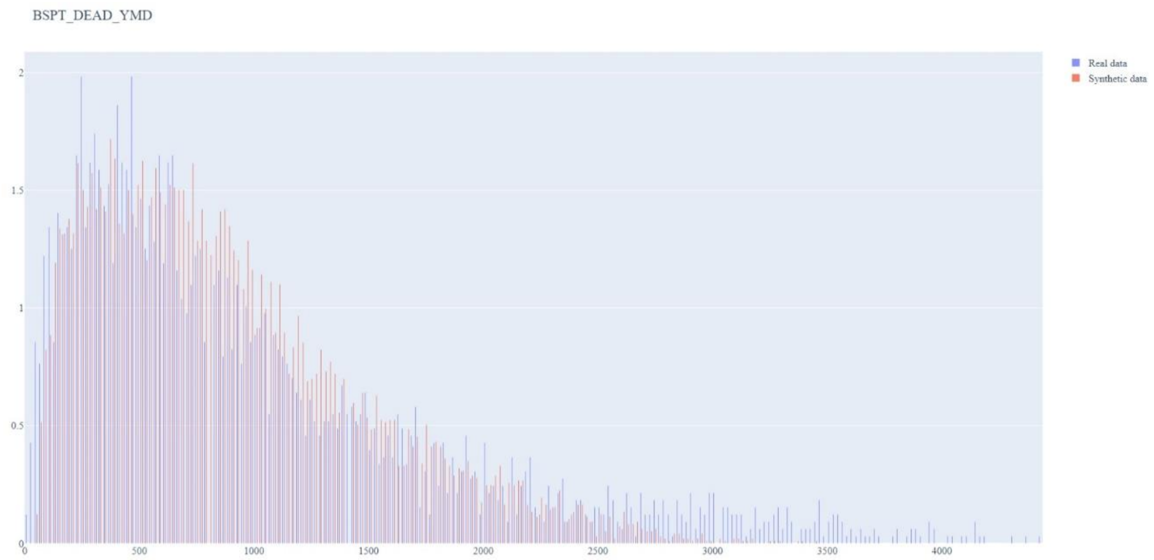

Figure 5: Histogram of date of death between real and synthetic data

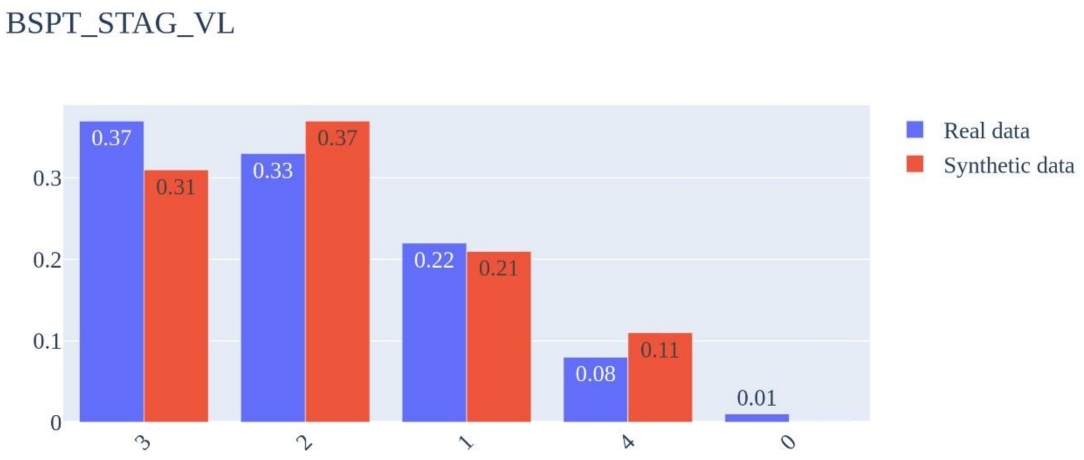

Figure 6: Histogram of patient staging value between real and synthetic data

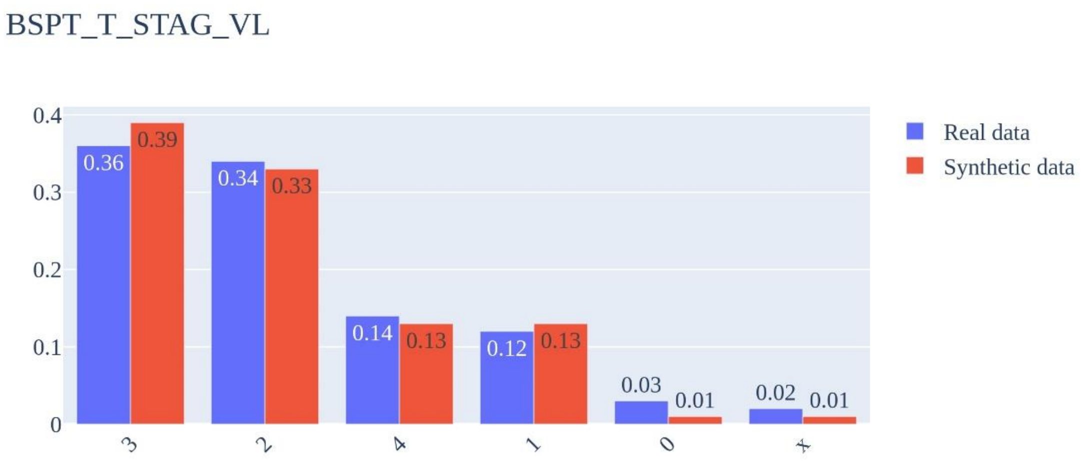

Figure 7: Histogram of patient staging value T between real and synthetic data

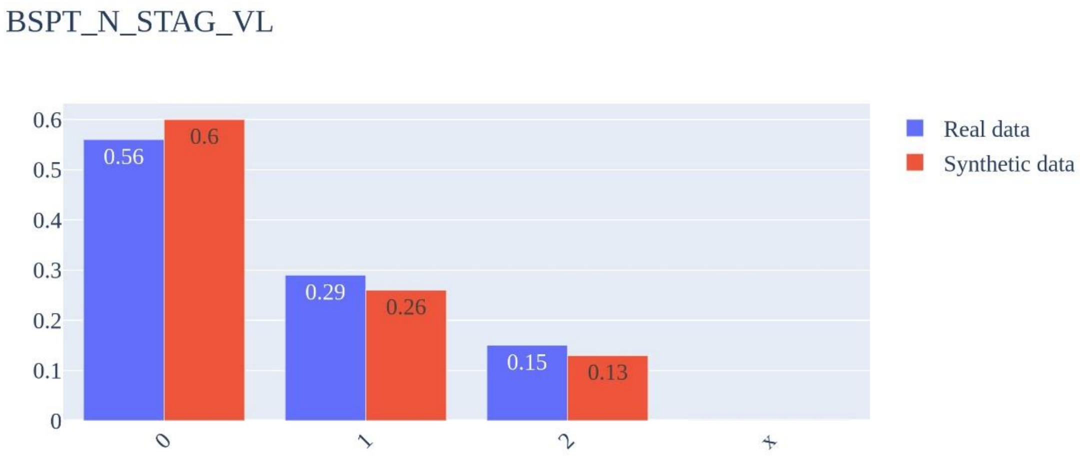

Figure 8: Histogram of patient staging value N between real and synthetic data

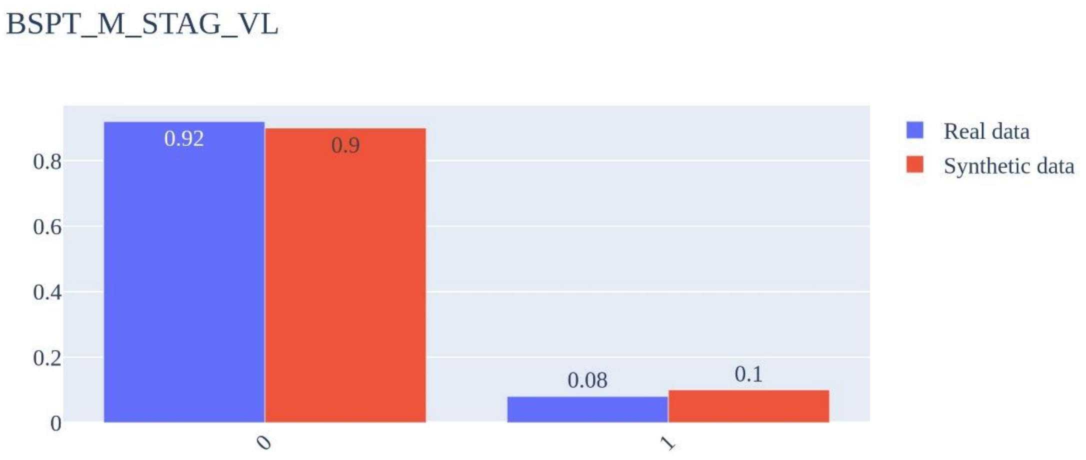

Figure 9: Histogram of patient staging value M between real and synthetic data

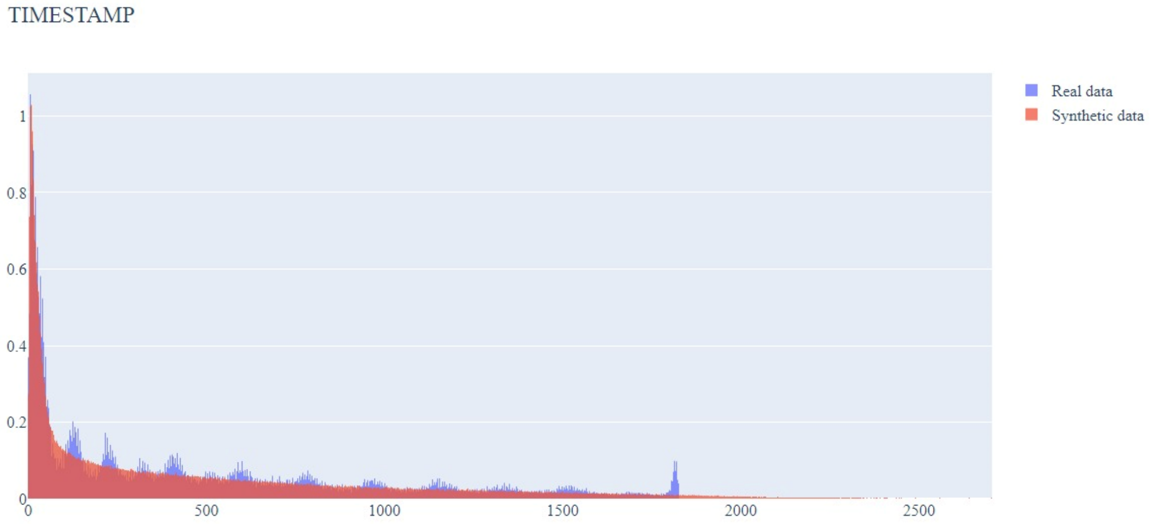

Figure 10: Histogram of patient timestamp between real and synthetic data

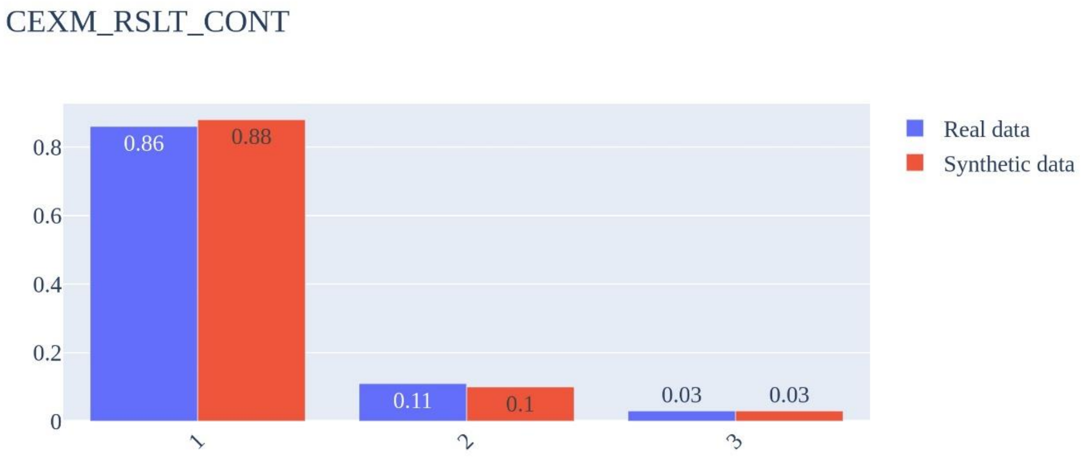

Figure 11: Histogram of diagnostic test results between real and synthetic data

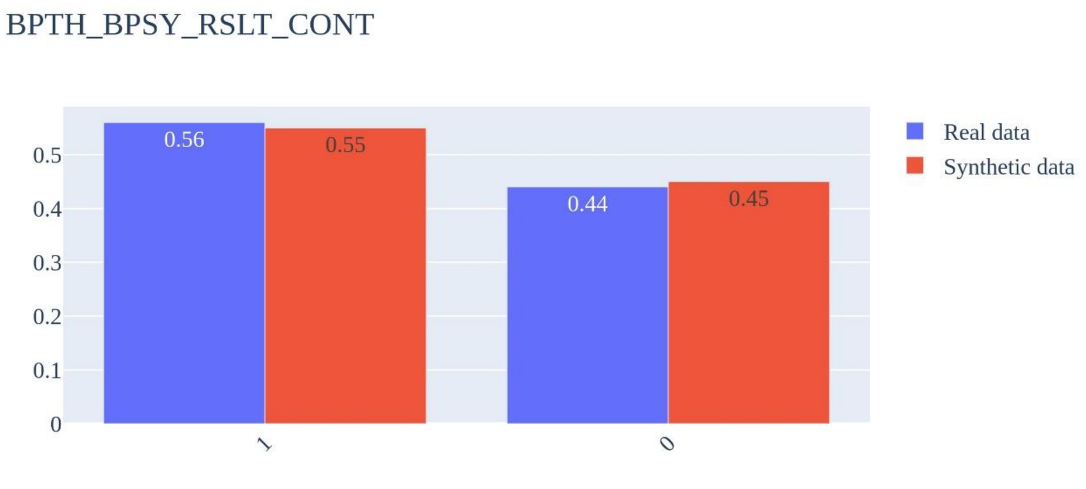

Figure 12: Histogram of biopsy results between real and synthetic data

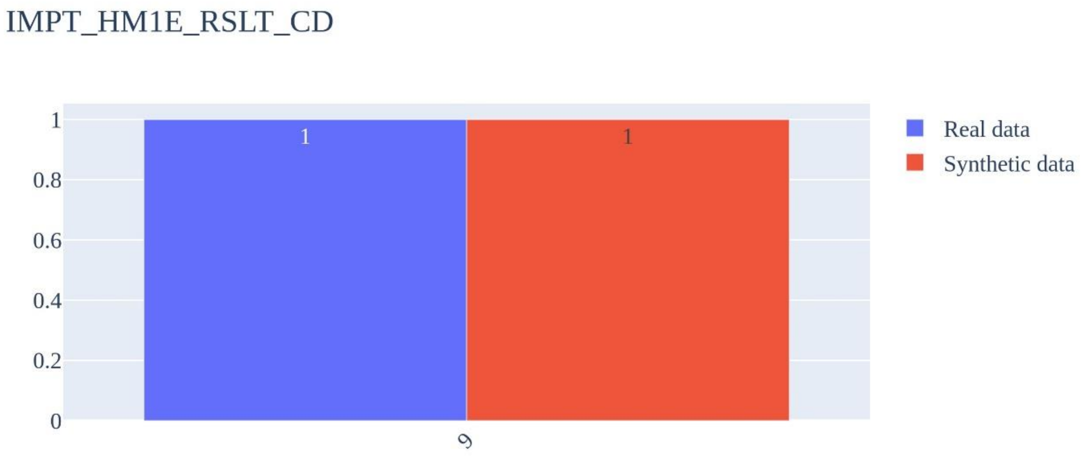

Figure 13: Histogram of HM1E result code between real and synthetic data

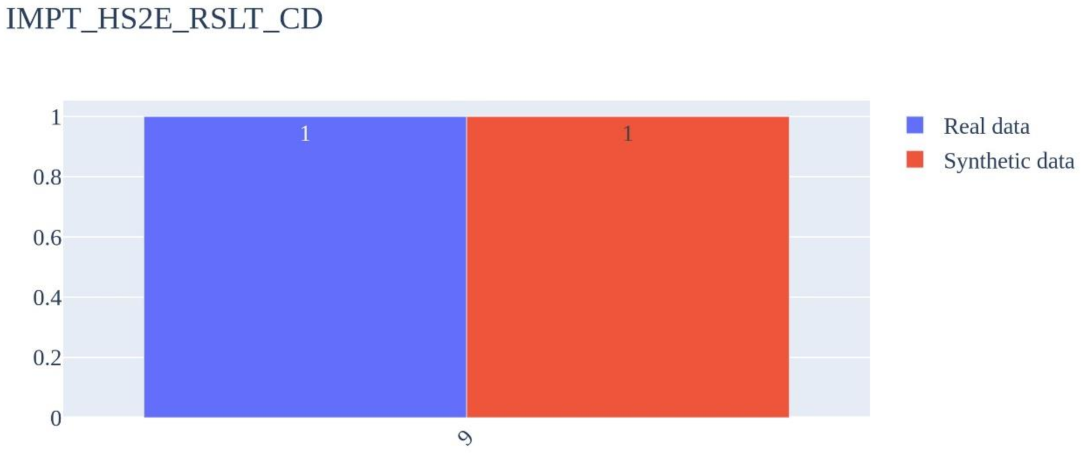

Figure 14: Histogram of HS2E result code between real and synthetic data

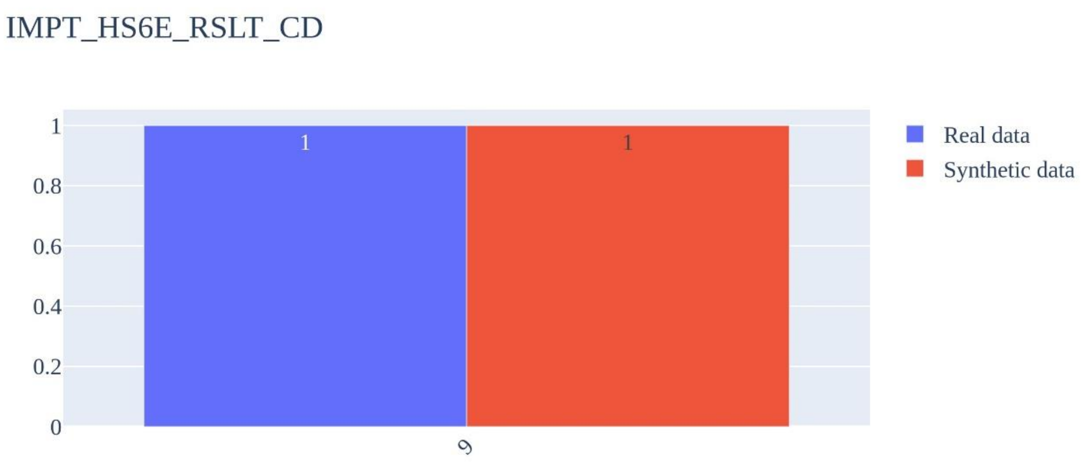

Figure 15: Histogram of HS6E result code between real and synthetic data

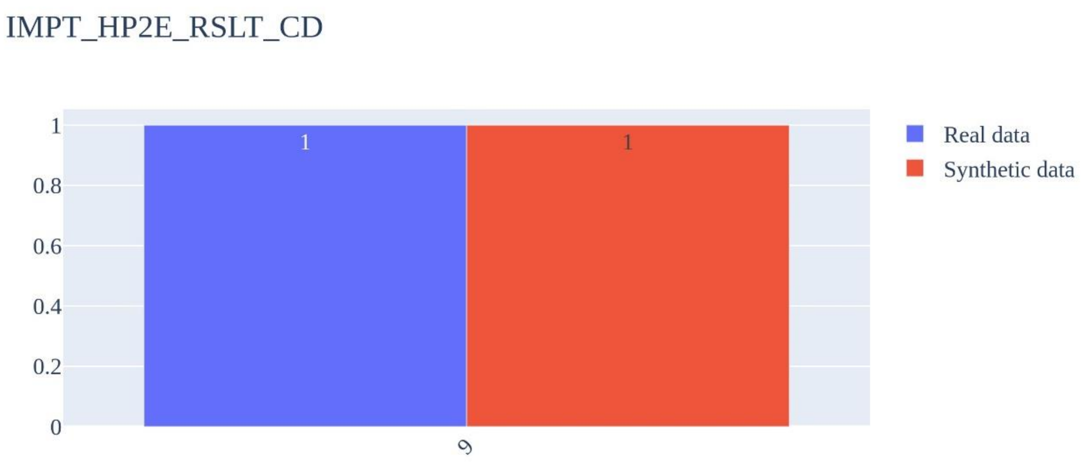

Figure 16: Histogram of HP2E result code between real and synthetic data

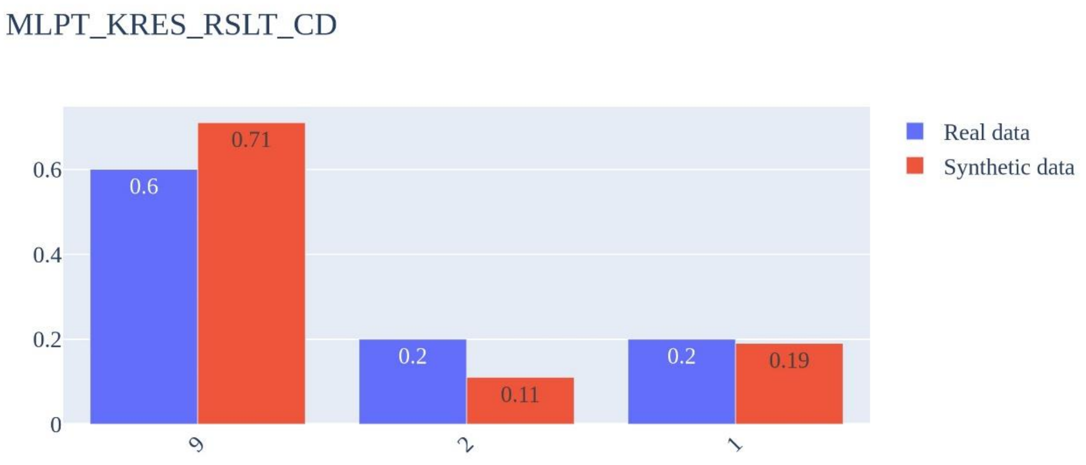

Figure 17: Histogram of KRASMUTATION result code between real and synthetic data

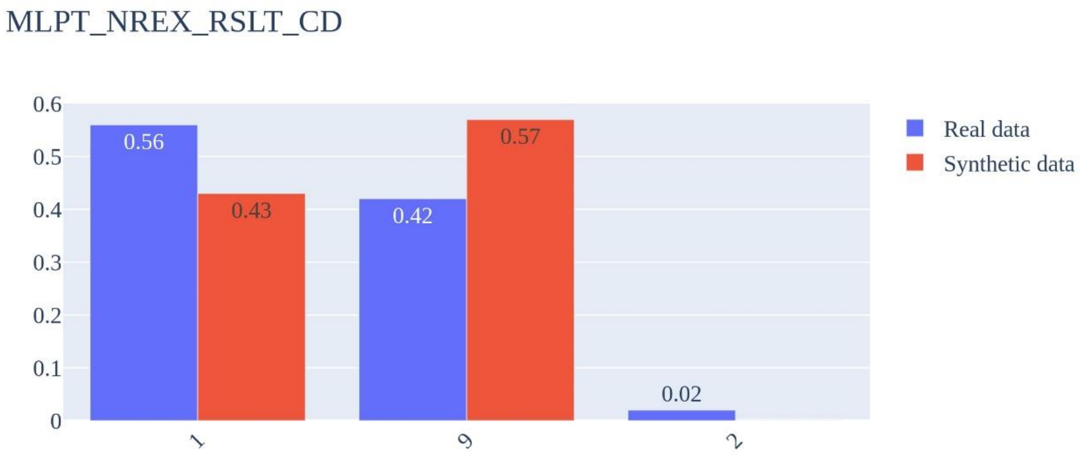

Figure 18: Histogram of NRASMUTATION result code between real and synthetic data

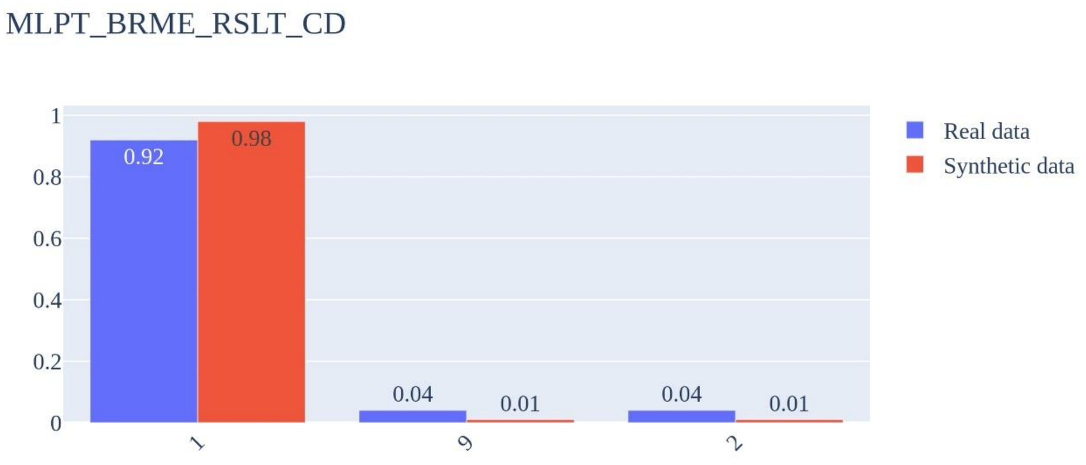

Figure 19: Histogram of BRAFMUTATION result code between real and synthetic data

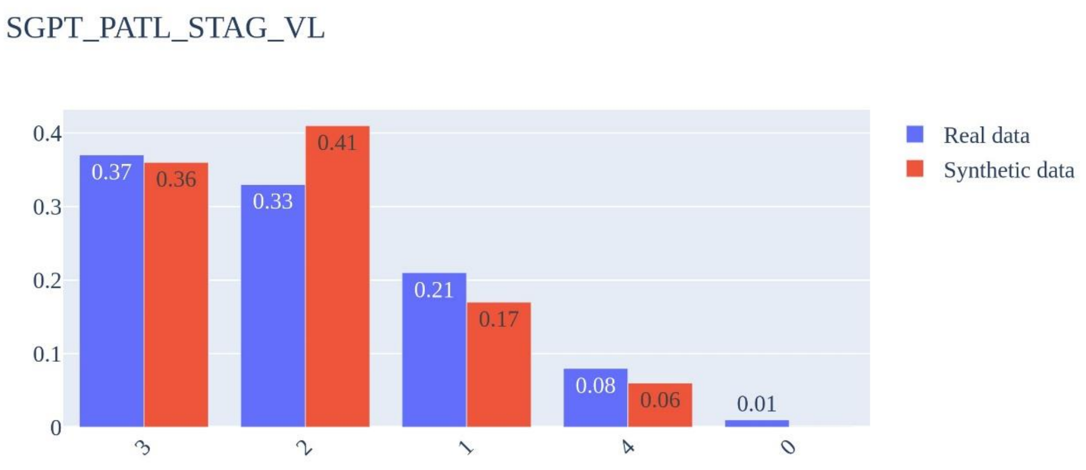

Figure 20: Histogram of staging value between real and synthetic data

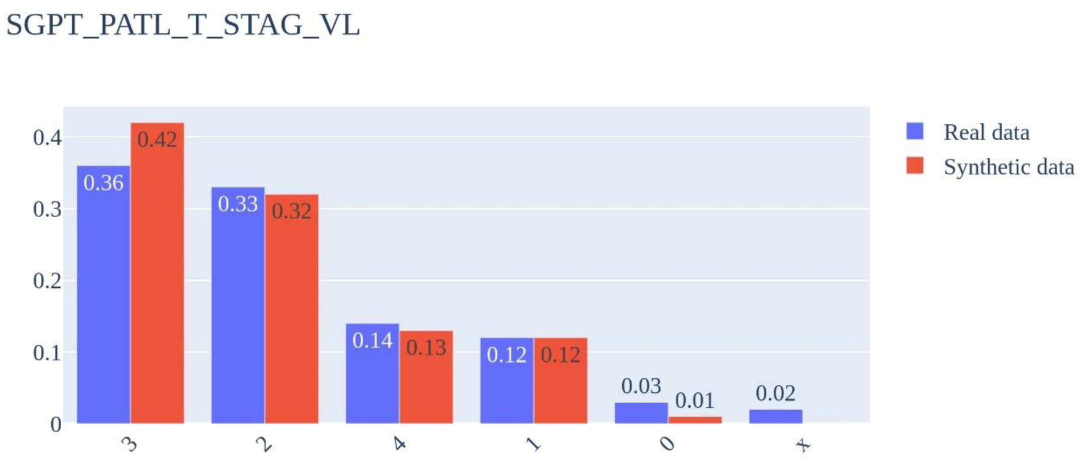

Figure 21: Histogram of staging value T between real and synthetic data

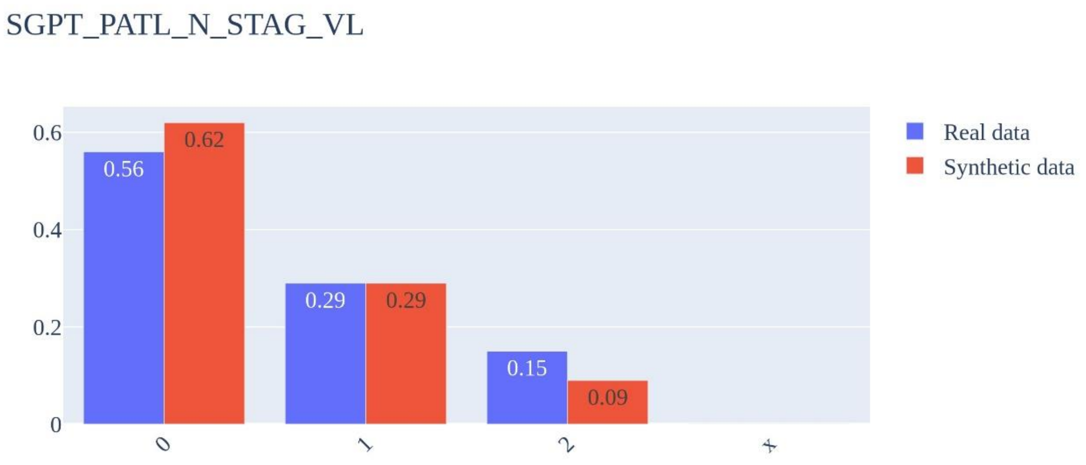

Figure 22: Histogram of staging value N between real and synthetic data

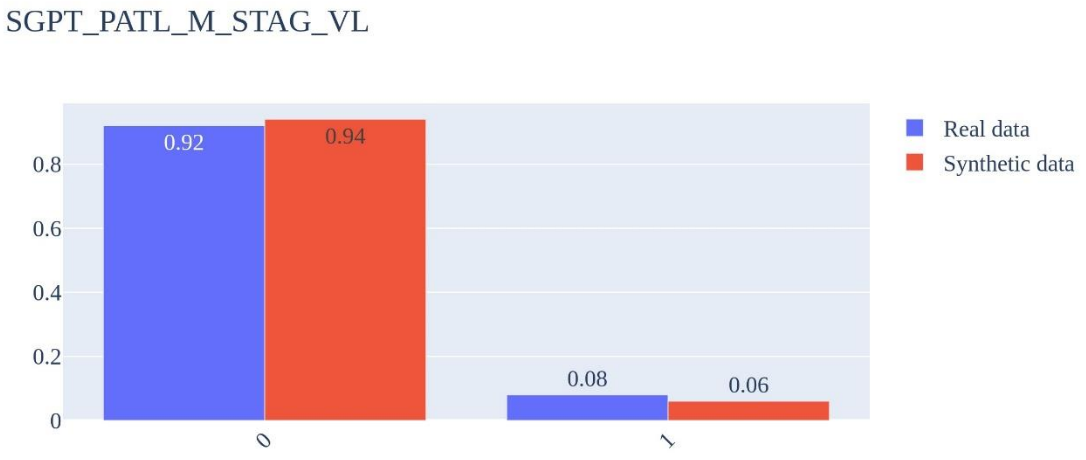

Figure 23: Histogram of staging value M between real and synthetic data

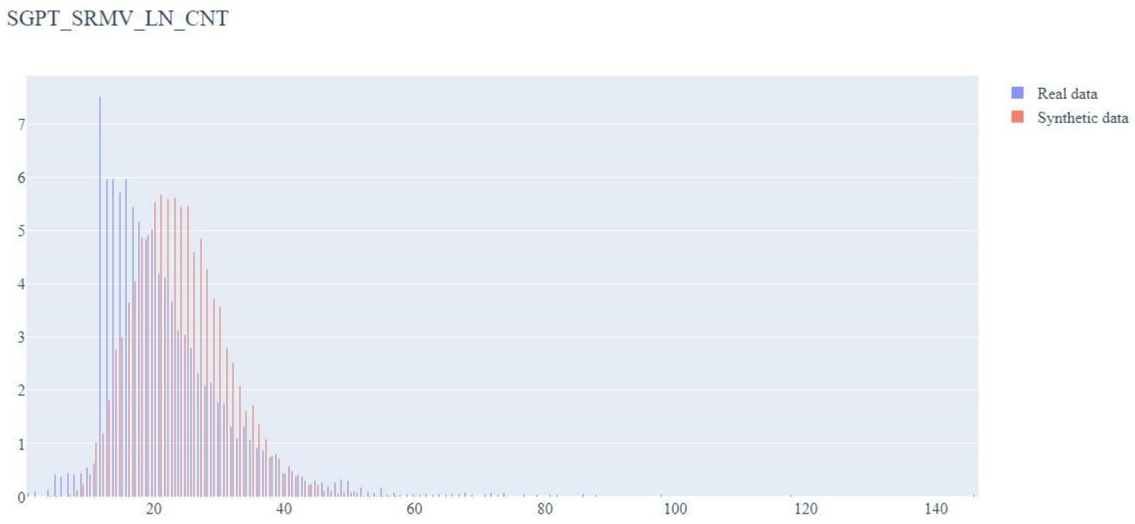

Figure 24: Histogram of resected lymph nodes between real and synthetic data

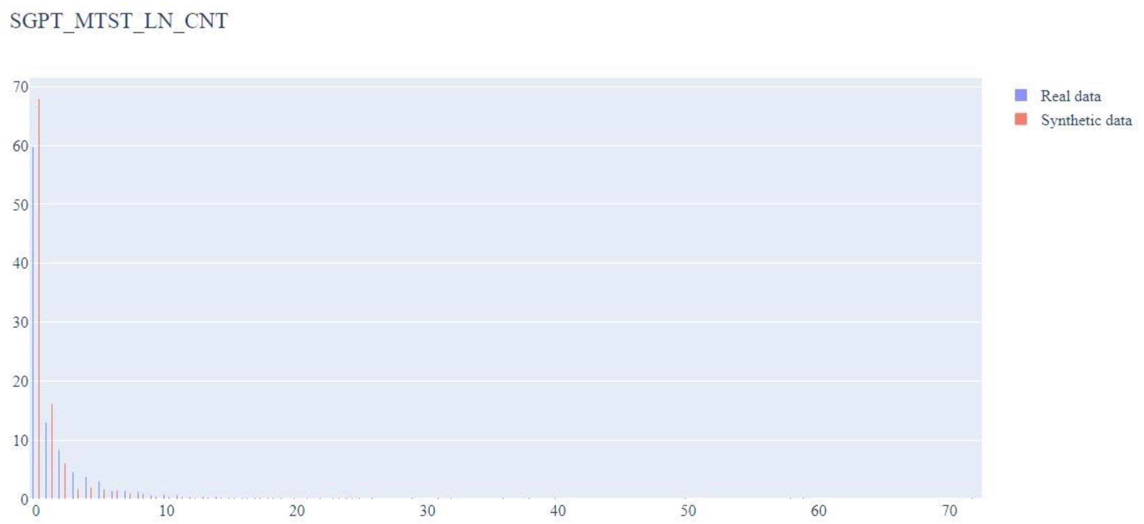

Figure 25: Histogram of metastatic lymph node between real and synthetic data

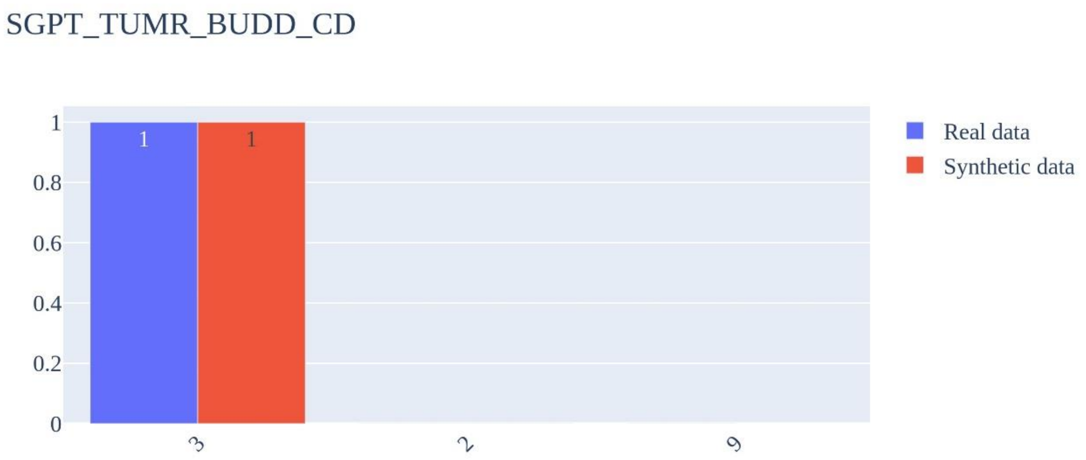

Figure 26: Histogram of surgical resection surface distal cancer tissue size status code between real and synthetic data

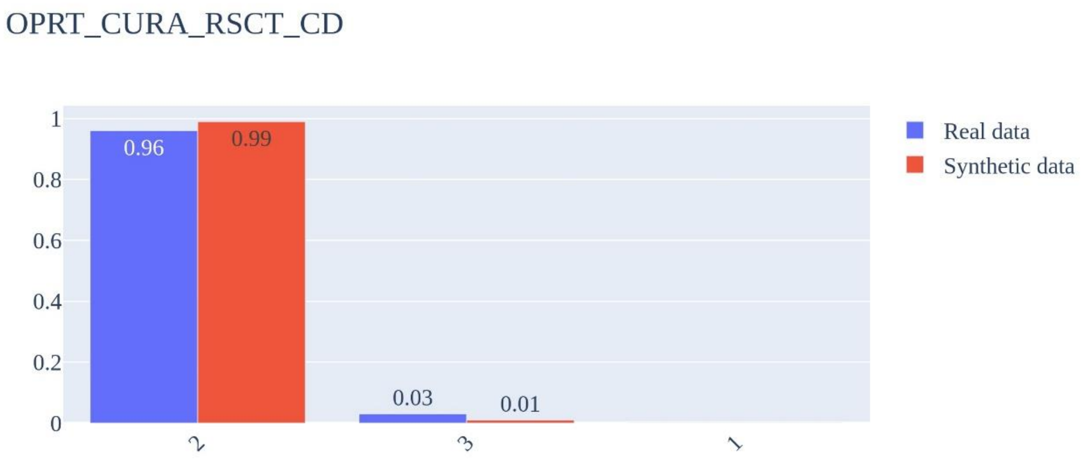

Figure 27: Histogram of radical resection code between real and synthetic data

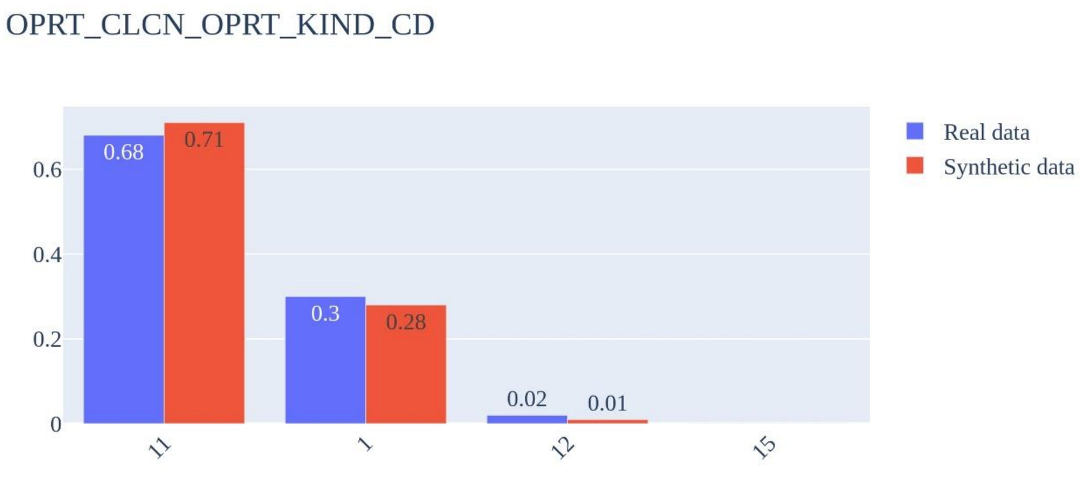

Figure 28: Histogram of colorectal cancer surgery type code between real and synthetic data

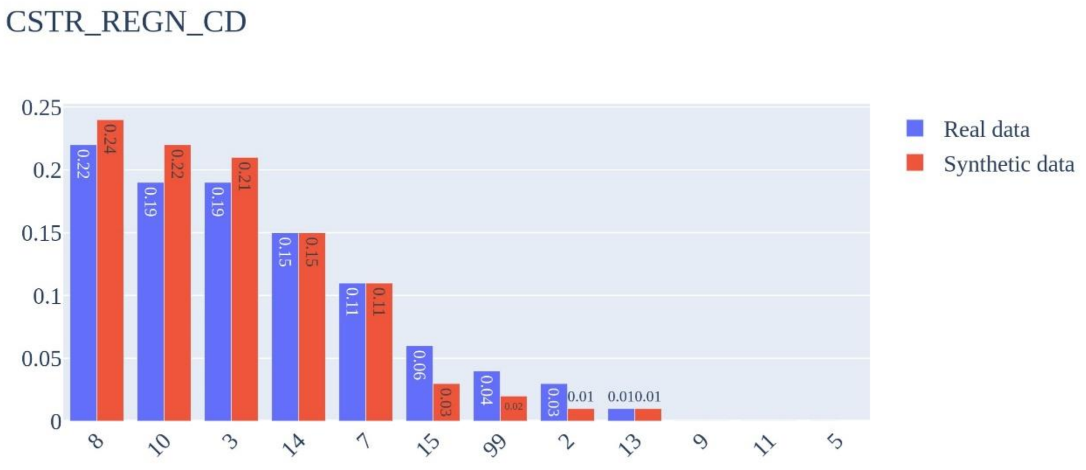

Figure 29: Histogram of anticancer drug treatment code between real and synthetic data

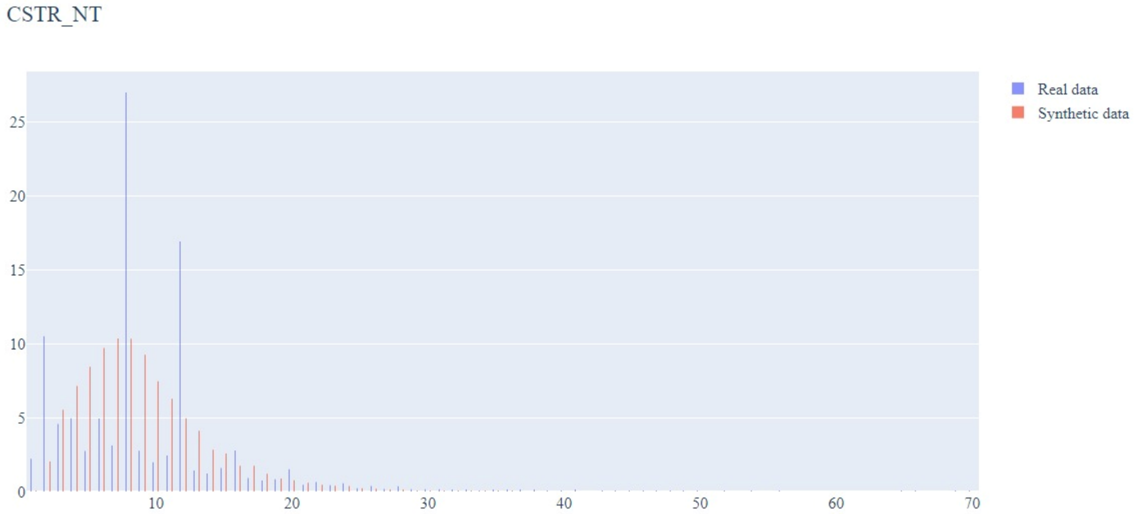

Figure 30: Histogram of number of chemotherapy treatments between real and synthetic data

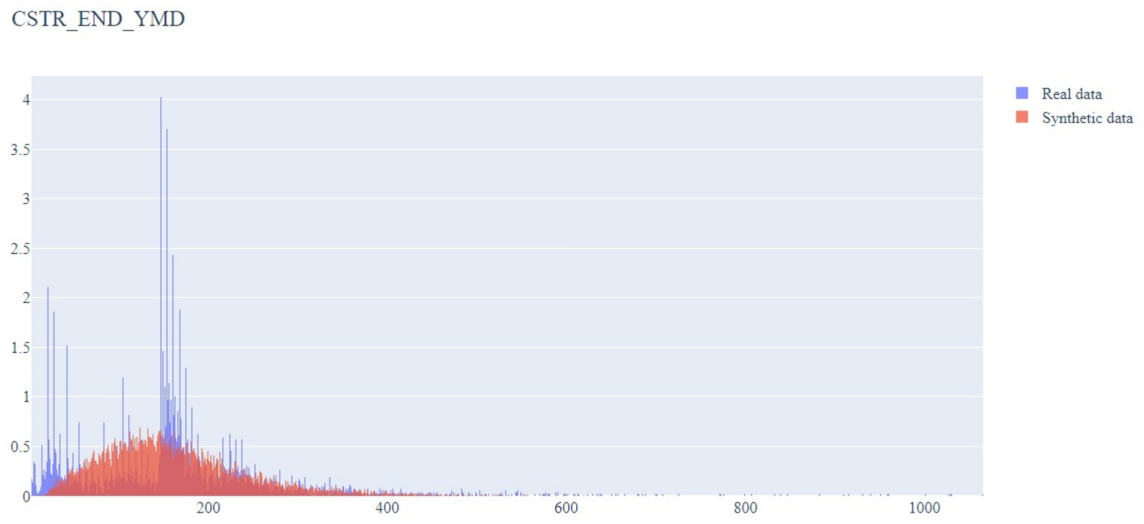

Figure 31: Histogram of Anticancer drug treatment end date between real and synthetic data

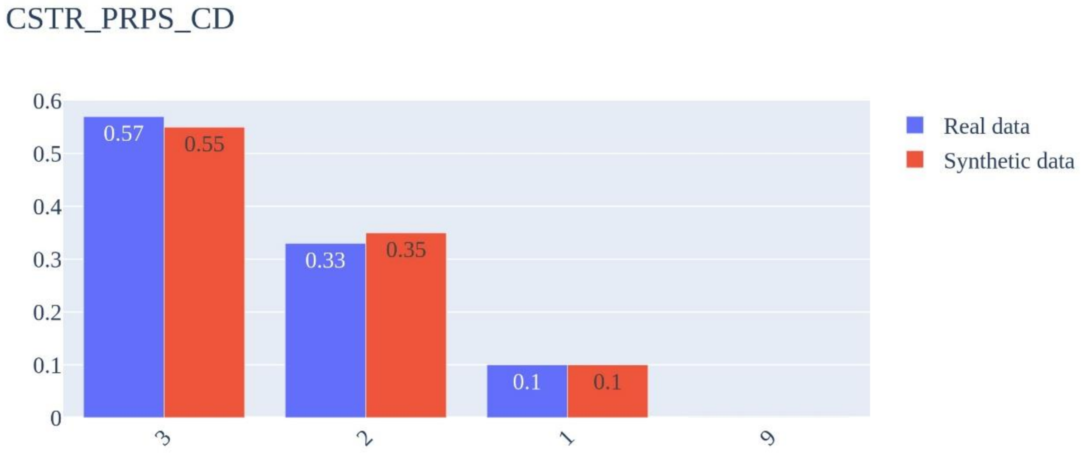

Figure 32: Histogram of Anticancer drug treatment purpose code between real and synthetic data

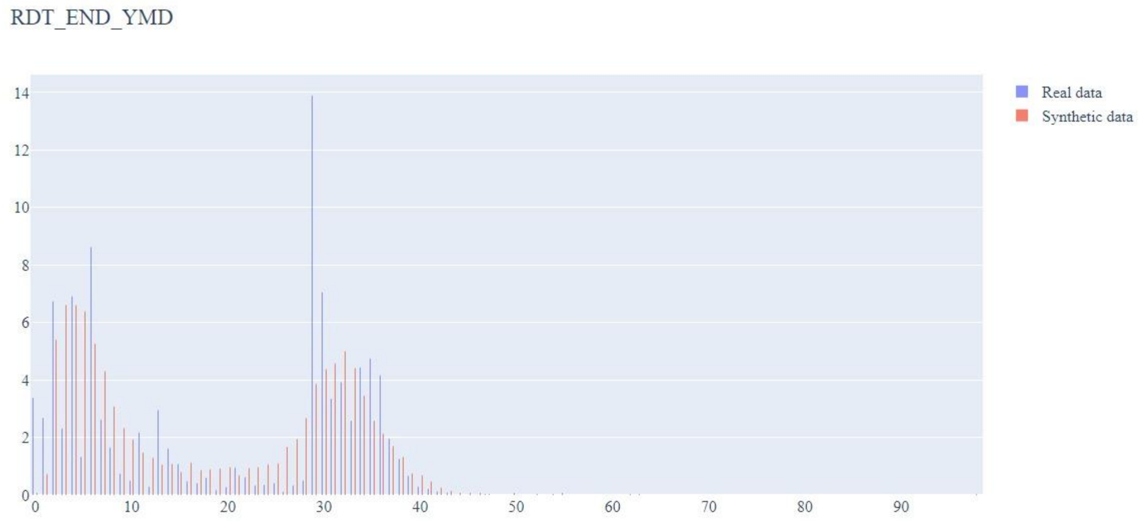

Figure 33: Histogram of Radiation treatment completion date between real and synthetic data

1. K. El Emam, L. Mosquera, X. Fang, A. El-Hussuna, Utility metrics for evaluating synthetic health data generation methods: validation study. *JMIR medical informatics* **10**, e35734 (2022).
